# Modulation of Oxidative Stress: Pharmaceutical and Pharmacological Aspects 2018

**DOI:** 10.1155/2019/6380473

**Published:** 2019-07-03

**Authors:** Robert E. Smith, Tomris Ozben, Luciano Saso

**Affiliations:** ^1^Park University, 8700 River Park Drive, Parkville, MO 64152, USA; ^2^Department of Biochemistry, Akdeniz University Medical Faculty Campus, Dumlupinar Street, 07070 Antalya, Turkey; ^3^Department of Physiology and Pharmacology “Vittorio Erspamer”, Sapienza University of Rome, Piazzale Aldo Moro 5, 00185 Rome, Italy

Chronic, low-grade, smoldering inflammation (also called parainflammation) can lead to cardiovascular and neurodegenerative diseases, as well as many types of cancer [1]. It can be caused by obesity, metabolic syndrome, and even periodontal disease. One of the articles in this special issue tells how adding antioxidants to dental materials can help to prevent oxidative stress in gingival fluid (“Influence of Dental Restorations on Oxidative Stress in Gingival Crevicular Fluid” (E. Taso et al.)).

Still, inflammation has been one of the most misunderstood aspects of health when viewed with reductionist thinking [1, 2]. Part of the misunderstanding was based on the assumption that the effects of inflammation on model organisms such as baker's yeast (*Saccharomyces cerevisiae*), the fruit fly *Drosophila melanogaster*, mice, rats, and dogs can tell us what also happens in humans. That is, caloric restriction without starvation has extended the lifespans of these organisms. It was thought that restricting the consumption of calories by reducing the consumption of proteins, fats, and carbohydrates reduced the total metabolic rate and the production of reactive oxygen and nitrogen species (RONS) and free radicals. This led to the free radical theory of aging, in which it was proposed that the accumulation of damage caused by free radicals causes aging and eventually death. Note that most authors use the term ROS (reactive oxygen species), instead of RONS. Still, some ROS or RONS (like nitric oxide or NO) have a reactive nitrogen, rather than a reactive oxygen. This free radical theory encouraged people to eat foods that have a high antioxidant capacity. So, *in vitro* tests for total antioxidant capacity emerged. They were based on measuring the destruction of oxidized test compounds by reacting directly with the antioxidants in foods [1, 2].

However, as scientists and physicians learned more about human nutrition, they realized that the caloric restriction that worked for other organisms did not work for humans [1, 2]. This required changing the paradigm from reductionist thinking to systems thinking. It is important to use systems thinking and realize that inflammation can be not just a cause or a symptom of many diseases, but it is also essential for good health. Inflammation, like so much else in the body, must be carefully controlled [1, 2].

Even though vitamins A and C as well as CoQ10 can react directly with RONS and destroy them, other antioxidants seldom do this [1, 2]. Most antioxidants (especially phenolic compounds) do not work by reacting directly with RONS and free radicals. They exert their health effects by first activating the transcription factor nuclear erythroid 2 like factor-2 (Nrf2), which then induces the transcription of endogenous antioxidant response elements (AREs). Nrf2 controls the expression of AREs by binding to their promoter regions. The combined system is called the Nrf2/ARE antioxidant signaling system [1, 2].

The Nrf2/ARE signaling system can prevent cardiovascular disease (CVD) by preventing smoldering inflammation [1–3]. It also helps prevent neurodegenerative diseases and limit the damage caused by ischemia during a stroke. One of the articles in this issue (“C_60_ Fullerene Prevents Restraint Stress-Induced Oxidative Disorders in Rat Tissues: Possible Involvement of the Nrf2/ARE-Antioxidant Pathway” (O. O. Gonchar et al.)) describes how a type of fullerene (also known as Buckminsterfullerenes and Buckyballs) containing 60 carbons (C_60_ fullerene) can prevent stress-induced oxidative disorders by activating the Nrf2/ARE antioxidant system. It is interesting to note that C_60_ fullerene has two opposite properties regarding RONS or ROS [4]. When exposed to visible light, it can produce ROS, making it suitable for photodynamic therapy. It can also downregulate the production of ROS in cells, making a neuroprotective agent. It can also be used as a drug delivery system. In one system, C_60_ fullerene was conjugated with the popular and effective anticancer drug, doxorubicin. C_60_ fullerene is used for targeted drug delivery to reduce the cardiomyopathy that occurs frequently when doxorubicin is used without C_60_ fullerene. It also breaks links that are sensitive to ROS, enabling the release of doxorubicin into cancer cells [4].

A completely different substance, uric acid, was shown to protect against the deadly effects of oxidative stress caused by ischemia-reperfusion, as described in another article in this issue (“Uric Acid Protects against Focal Cerebral Ischemia/Reperfusion-Induced Oxidative Stress via Activating Nrf2 and Regulating Neurotrophic Factor Expression” (B. Ya et al.)). It provides this protection by activating the Nrf2/ARE system, which was already known to play a critical role in ischemic stroke [5].

Another article in this issue (“Modulation of Hippocampal Antioxidant Defense System in Chronically Stressed Rats by Lithium” (N. Popović et al.)) tells how a widely used drug to treat bipolar disorders (lithium) exerts its therapeutic effects, in part by increasing the activity of some of the enzymes that are activated by Nrf2. This reduces the oxidative stress in the hippocampus [5]. This adds another mechanism to the previously described ability of lithium to inhibit inositol monophosphatase and glycogen synthase kinase-3 [6, 7].

Oxidative stress is also prevalent in children suffering from autism spectrum disorder (ASD). It alters the shape of erythrocytes and reduces oxidative stress (“Oxidative Stress in Autistic Children Alters Erythrocyte Shape in the Absence of Quantitative Protein Alterations and of Loss of Membrane Phospholipid Asymmetry” (A. Bolotta et al.)). Oxidative stress is reduced due to the activity of the antioxidant enzyme peroxiredoxin-2 (Prx2), which is upregulated by Nrf2 [1–3].

Not only the brain but also the eyes can be affected by oxidative stress, as described in another article in this series (“Sodium Ferulate Attenuates Lidocaine-Induced Corneal Endothelial Impairment” (G. Jiang and T. Fan)). When lidocaine is used as an anesthetic in cataract surgery, it can cause corneal thickening, opacification, and loss of corneal endothelial cells. When sodium ferulate is included, the toxicity of lidocaine is reduced. Ferulate activates the Nrf2/ARE system [1–3].

Oxidative stress can also affect stem cells. Another article in this series (“Pharmacological Regulation of Oxidative Stress in Stem Cells” (J. Lee et al.)) describes how the redox state of a stem cell affects the balance between self-renewal and differentiation. Hematopoietic stem cells (HSCs) are adult stem cells that form blood cells that are needed to replace the ones that die. They stay in hypoxic niches in the bone marrow, which protects them from oxidative stress. As a result, they can maintain their ability to self-renew. HSCs need to be protected from excess ROS but need continuous ROS production at low levels to maintain their biological function. Since stem cells are also important in replacing damaged tissues in regenerative medicine, attempts are being made to regulate oxidative stress in them through pharmacology. There are radioprotective substances and drugs that can protect stem cells in the liver from injury due to the use of radiation therapy. Melatonin, alpha-lipoic acid, and conjugated 5-methoxytryptamine-*α*-lipoic acid decrease the concentration of ROS in hematopoietic cells by inhibiting NADPH oxidase 4 (NOX4), which is upregulated by the Nrf2/ARE system. There is also an FDA-approved prescription drug, amifostine, that is a ROS scavenger and radioprotective drug. In addition, cyclosporine A inhibits ROS production that is linked to cyclophilin D. This increased the yield of hematopoietic stem cells obtained from the bone marrow and cord blood.

Alpha-lipoic acid is a popular dietary supplement [8–10]. It is used to treat diabetic polyneuropathy, obesity, and related metabolic disorders, as well as to help prevent vascular disease, hypertension, and inflammation. When given in combination with L-carnosine, zinc, and B vitamins, alpha-lipoic acid improved fasting glucose and insulin resistance and decreased the level of glycosylated hemoglobin (Hb_A1C_) [11]. However, in an article in this issue (“The Effect of 600 mg Alpha-lipoic Acid Supplementation on Oxidative Stress, Inflammation, and RAGE in Older Adults with Type 2 Diabetes Mellitus” (V. M. Mendoza-Núñez et al.)), alpha-lipoic acid by itself (at a dose of 600 mg/day for six months) did not have a significant antioxidant or anti-inflammatory effect.

Oxidative damage can also occur in the lungs, leading to respiratory diseases. One article in this series describes the role of the Nrf2/ARE system and compounds that activate it (“Role of Nrf2 and Its Activators in Respiratory Diseases” (Q. Liu et al.)). Activators of Nrf2 can help prevent bronchopulmonary dysplasia, respiratory infections, acute respiratory stress syndrome, chronic obstructive pulmonary disease (COPD), asthma, idiopathic pulmonary fibrosis, and lung cancer. Activated Nrf2 can also protect against infection by the respiratory syncytial virus (RSV), which decreases Nrf2 activity when the RSV is active. In addition, sulforaphane in broccoli, curcumin in turmeric, and epigallocatechin gallate (EGCG) in green tea can help to protect against damage that is done by infection by the influenza A virus.

The use of a tourniquet can cause damage due to ischemia-reperfusion. One of the articles in this issue (“The Possible Pathophysiological Outcomes and Mechanisms of Tourniquet-Induced Ischemia-Reperfusion Injury during Total Knee Arthroplasty” (P. Leurcharusmee et al.)) tells how a mixture of ischemic preconditioning, vitamin C, and propofol (a popular general anesthetic) protected against oxidative and inflammatory damage.

Infection by the hepatitis C virus leads to oxidative stress in the acute and persistent phases of infection, as described in another article in this series (“Counteraction of HCV-Induced Oxidative Stress Concurs to Establish Chronic Infection in Liver Cell Cultures” (S. Anticoli et al.)). On the other hand, a reduced environment emerges during the chronic phase of infection, as the concentration of ROS decreases and the activity of the antioxidant enzyme, glutathione reductase, increases.

In another article in this series, flavonoids from silymarin (a seed extract of *Silybum marianum* (L.) Gaertn.) were shown to increase systemic and hepatic bilirubin concentrations and lower lipoperoxidation in mice (“Isolated Silymarin Flavonoids Increase Systemic and Hepatic Bilirubin Concentrations and Lower Lipoperoxidation in Mice” (J. Ŝuk et al.)). Even though bilirubin (the end product of heme catabolism) has been thought of as primarily a toxic catabolite and a sign of liver dysfunction, it is a potent antioxidant that has anti-inflammatory, antiproliferative, antigenotoxic, and antiaging properties. Bilirubin is also an agonist of the peroxisome proliferator-activated receptor-*α* (PPAR*α*), a master regulator of lipid metabolism that inhibits the development of atherosclerosis, plaque rupture, and thrombus formation [12].

In another article in this series, a new potential antitumor agent and coumarin derivative was synthesized and characterized (“Synthesis and Characterization of 3-(1-((3,4-Dihydroxyphenethyl)amino)ethylidene)-chroman-2,4-dione as a Potential Antitumor Agent” (D. S. Dimić et al.)). It was active against carcinoma cell lines, especially the MCF7 breast carcinoma.

In conclusion, this issue contains a variety of articles about the pharmaceutical and pharmacological aspects of the modulation of oxidative stress.
